# Range of surgical strategies for individual adolescent idiopathic scoliosis cases: evaluation of a multi-centre survey

**DOI:** 10.1007/s43390-023-00756-0

**Published:** 2023-08-28

**Authors:** Benedikt Schlager, Maresa Großkinsky, Michael Ruf, Bernd Wiedenhöfer, Michael Akbar, Hans-Joachim Wilke

**Affiliations:** 1 Institute of Orthopaedic Research and Biomechanics, Centre for Trauma Research Ulm, Ulm University Medical Centre, Helmholtzstraße 14, 89081 Ulm, Germany; 2 SRH Klinikum Karlsbad-Langensteinbach, Karlsbad, Germany; 3 ATOS Klinik Heidelberg GmbH & Co. KG, Heidelberg, Germany; 4 MEOCLINIC, Berlin, Germany

**Keywords:** Adolescent idiopathic scoliosis, Orthopaedics, Surgery, Biomechanics, Survey

## Abstract

**Purpose:**

Surgical treatment of adolescent idiopathic scoliosis (AIS) is very complex, involves many critical decisions and modern instrumentation techniques, and offers multiple possibilities. It is known that the surgical strategy may vary strongly between surgeons for AIS cases. The goal of this study was to document, summarize, and analyse the current biomechanical relevant variabilities in the surgical treatments of individual AIS patient cases.

**Methods:**

Eight experienced scoliosis surgeons from different hospitals were asked to plan surgeries on 12 representative patients with AIS. The surgeons were provided with radiographs during upright standing in the coronal and sagittal plane, as well as lateral bending images to the left and right. The surgeons were asked to specify the Lenke type, their surgical approach, the resection steps, the planned fusion length, and the type of implants. The data were analysed with respect to the inter-rater variability, which was quantified using the Fleiss Kappa method.

**Results:**

In the selection of the surgical approach, the surgeons concurred most with Lenke curve types 2 (*κ = *0.88) and 4 (*κ = *0.75). The largest differences were shown at Lenke 1 (*κ = *0.39) and 5 (*κ = *0.32). Anterior approaches were selected in the majority of cases at Lenke types 5, with an average of 50%. The strongest deviation in fusion length was documented at Lenke curve type 6.

**Conclusion:**

The survey highlighted differences in the surgical strategy depending on the Lenke curve type, the direction of the surgical approach, and the surgeon. The main discrepancies between the surgeons were found for Lenke 1, 5, and 6 curves, and consistencies for Lenke 2, 3, and 4. The documented discrepancies indicate the remaining open questions in the surgical treatment and understanding of scoliosis biomechanics.

## Introduction

Adolescent idiopathic scoliosis (AIS) is a three-dimensional deformity of the spine with an incidence of about 0.5–5.2% amongst children [7]. While AIS is initially treated conservatively, surgical intervention is indicated for a progressive deformity above 40° Cobb angle [4]. Surgical correction is performed by conducting a spinal fixation to stabilize the curvature and enable a bony fusion of the treated spinal curvatures.

Surgical treatment of adolescent idiopathic scoliosis (AIS) involves many critical decisions and modern instrumentation techniques offer multiple possibilities, such as the direction of the approach (from anterior or posterior), performed resection steps to mobilize the spine, amount of fused spinal segments, and type of implants.

Due to the variety of spinal deformities and the complex pathology, classifications and guidelines have been introduced on how to instrument the spine. King et al. grouped the AIS within five types of deformity according to the location of the main curvatures [6]. Lenke et al. further refined the scoliosis classification by grouping AIS into six types of deformities by considering the location of the main deformity, the flexibility of the curvatures, the lumbar modifier, as well as sagittal profile [10].

A survey with 32 North American surgeons published in 2007 revealed that a great inter- and intra-rater variability exists amongst surgeons [3, 12]. Within this study, the surgeons were asked to perform surgical correction on five representative AIS cases, which were all instrumented with a posterior fixation. The authors identified three groups of surgeons: one which used pedicle screws only, one with hooks only, and a third group which used hooks and screws.

Erken et al. even found disagreements in surgical planning within a single centre between four surgeons planning the same adolescent idiopathic scoliosis cases [5]. These disagreements were found in 31% of 100 treated patients, while mainly the selection of the upper (UIV) and lower (LIV) instrumented vertebrae varied.

In an international survey published in 2013, 48 surgeons were asked to specify what belonged to an optimal AIS surgical treatment [4]. The authors obtained an average consensus within 70% of all surgeons. Consent was documented especially within the required pre-operative images, the selection of the instrumentation, as well as the mobilization using Ponte osteotomies at strong deformations.

In the last decade, surgical approaches and instrumentation techniques have evolved.

The goal of this study was to document and analyse the current surgical strategies for AIS patients in Germany for all Lenke curve types. This data was further used as reference for biomechanical studies to investigate the influence of different surgical strategies.

## Method

To document and analyse the current surgical strategies of AIS patients, a questionnaire was developed and sent to experienced scoliosis surgeons. The study involved eight experienced scoliosis surgeons from different scoliosis centres within Germany, with at least 5 years of experience and an operation rate of 25 AIS cases a year. To be able to evaluate and compare surgical strategies between the surgeons, the surgeons were asked to perform their surgical planning on the 12 representative AIS cases and document the strategies using a developed questionnaire. The representative AIS cases included ten female and two male AIS patients with an age range from 14 to 20 years. The aim was to represent all Lenke curve types 1–6 within the cases.

### The questionnaire

The questionnaire was designed to cover the main biomechanical aspects for the surgical treatment. In addition, the questionnaire needed to be simple to use, self-explaining, and to be completed fast to minimize the time load of the participating surgeons.

The questionnaire was implemented in a PDF format (Adobe Acrobat Pro, Adobe Inc.), which can be filled out on all prevalent computer systems, can be easily digitally distributed, and could potentially also be filled out analogue. The content of the questionnaire and its usability were verified together with two surgeons who tested the questionnaire.

The questionnaire included for each AIS patient case two sections. Within the first section, four radiographic images of the patient were presented, including the Cobb angles of the spinal curvatures in each plane. The radiographic images included the sagittal and coronal plane during upright standing, as well as in lateral bending to the left and right side in the supine position. Within these radiographic images, the spinal levels C7 to S1 were visible. The radiographic images were acquired retrospectively. Therefore, no radiographic images were taken explicitly for this study. In this part, the surgeons were asked to classify the scoliosis according to the Lenke classification system and select the direction of the surgical approach (anterior/posterior). Additionally, an empty field was available to add comments regarding the AIS case.

**Fig. 1:**
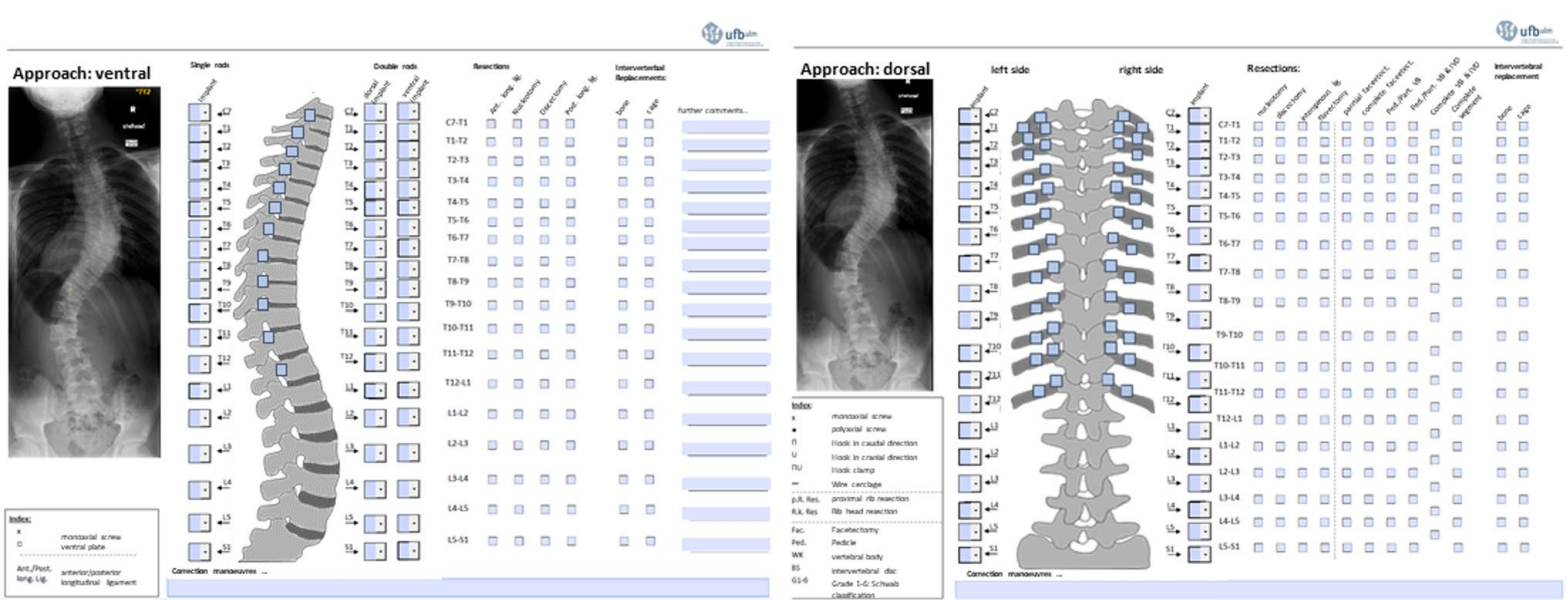
The questionnaire sheet to document the surgical planning for a right anterior (left) and posterior (right) approach. The surgical planning included the selection of the resected structures, the used implants on each side of a spinal level, and the intervertebral substitute. (The questionnaire was translated from German to English for the publication). Spine image modified from [13]

Within the second section, the surgeons were asked to specify the planned resection steps, the used implants for each vertebral segment on the left and right side, as well as the potential intervertebral disc substitute (Fig. 1). Resection steps included anterior and posterior ligaments, nucleotomy, discectomy, flavectomy, interspinous ligaments, as well as the resections according to Schwab grad 1–6: partial facetectomy (G1), complete facetectomy (G2), pedicle/partial vertebral body (G3), pedicle/partial vertebral body and disc (G4), total vertebra (G5), and whole segment (G6) resections. An additional option was the selection of level-specific rib-head resection on the left and right side.

The implant options, on each vertebral level and side, included ventral plate, mono-/polyaxial screws, hooks, and cerclage wire.

### Ethical concerns

In consultation with the ethical committee, no ethical vote was required for the survey. Each patient case was fully anonymised and the survey had no effect on the treatment of the patients.

### Evaluation

The data was evaluated in a descriptive way. The inter-rater reliability was quantified using the Fleiss’ Kappa statistic. Fields which were not selected were considered as “not relevant” and ignored in the case-specific evaluation.

## Results

All of the eight surgeons replied and documented their surgical planning using the questionnaire. Only in one AIS case, one surgeon could not perform a surgical planning based on the given information.

### Lenke classification

The surgeons’ classification according to Lenke resulted in a Fleiss Kappa value of 0.78 for the curve type, 0.87 of the lumbar modifier, and 0.87 for the thoracic sagittal modifier.

### Resection steps

**Fig. 2:**
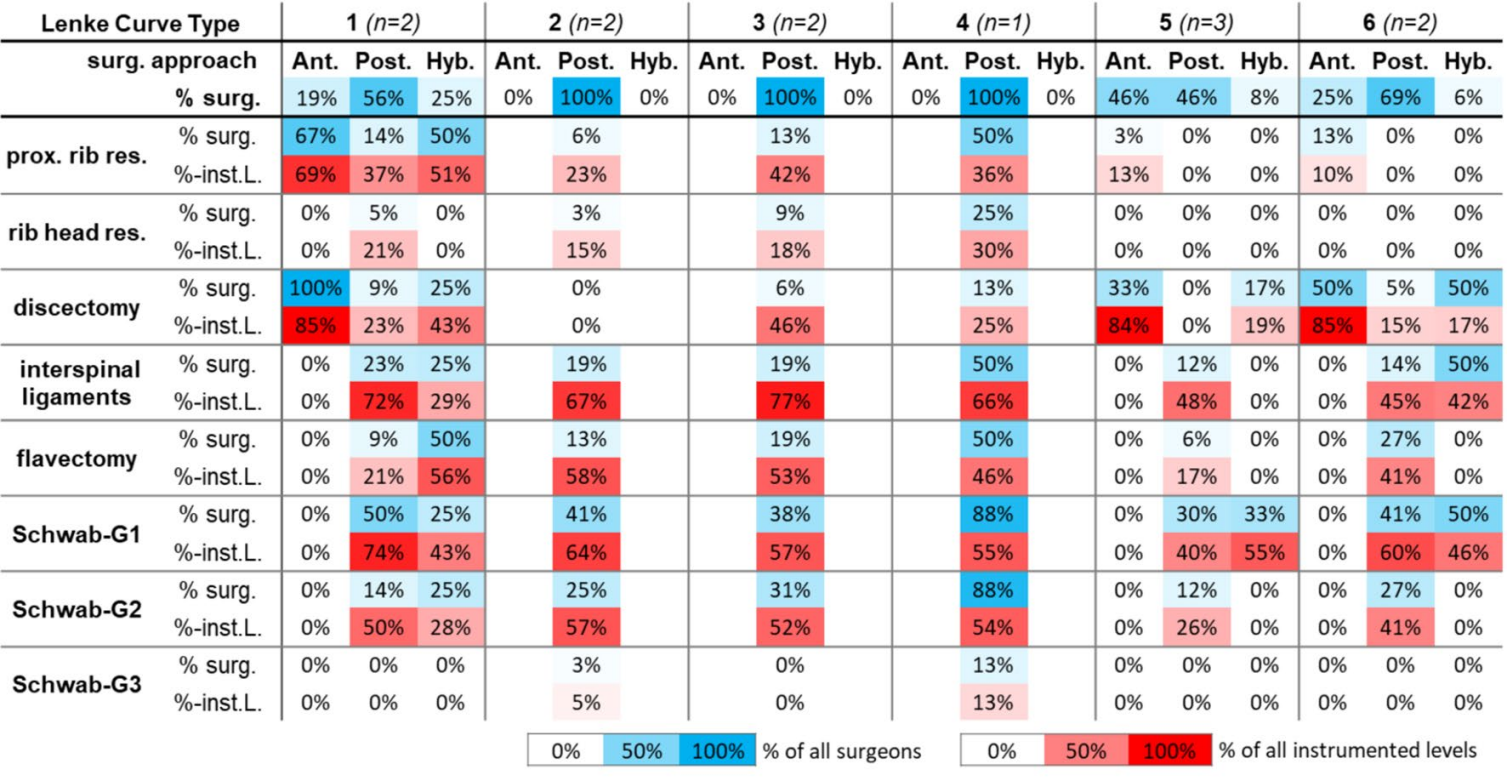
Planned resection steps for the spine and ribs, depending on the Lenke type and the direction of the surgical approach: anterior (ant), posterior (post), or hybrid (hyb, anterior and posterior). Example: “For Lenke 5 on average 46% of the surgeons selected an anterior approach. 33% of these surgeons planned a discectomy, at which a discectomy was planned on 84% of the discs relatively to the total fusion length.” *Inst. L* Instrumented Level; *prox* proximal, *Rib* ribs, *Res* resections, *surg* surgeons

The prevalent resection steps included discectomies, resections of the interspinous ligaments, and partial (Schwab G1) and complete facectomies (Schwab G2) (Fig. 2).

All surgeons who planned an anterior approach for Lenke 1 (3 out of 16, 19%) specified discectomies, with an average of 85% discectomies relative to the instrumented spinal region. One surgeon also planned discectomies within 46% of the instrumented levels in Lenke 3.

### Direction of the surgical approach

**Fig. 3:**
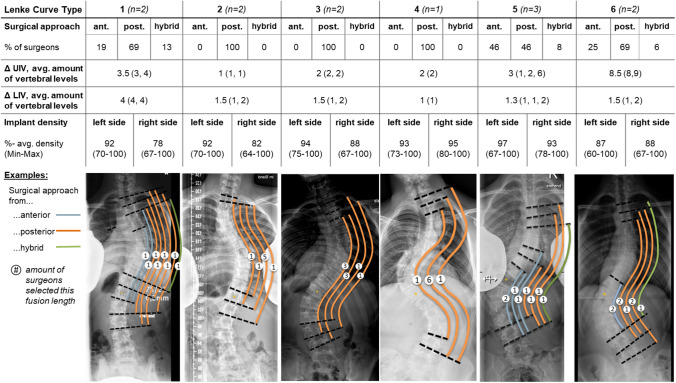
Surgical strategies to treat adolescent idiopathic scoliosis (AIS) depending of the Lenke curve type: percentage of surgeons who selected an anterior (ant.), posterior (post.), or ant. and post. (hybrid) approach, average deviation of the amount of upper instrumented vertebral (UIV) and lower instrumented vertebral (LIV) levels, as well as implant density on the left and right side of the spine. The below examples of AIS cases with the planned fusion length illustrated by coloured lines. The number within the circle indicates the amount of surgeons who planned this fusion length

All surgeons planed a posterior instrumentation for Lenke curve type 2, 3, and 4 (Fig. 3). The highest portions on anterior instrumentations were planned for Lenke 5 curve types, with 46% of the surgeons. One surgeon also planned an additional anterior approach for Lenke 3 and 4 to conduct discectomies.

### Fusion levels

The length of the instrumented levels varied for each Lenke curve type depending on the selection of the upper instrumented vertebra (UIV) and the lowest instrumented vertebra (LIV), as well as the direction of the approach (Fig. 3).

Strongest deviations were reported in the selection of the UIV for Lenke 6 with an average deviation of 8.5 spinal levels and 3.5 levels for Lenke 5. In Lenke 2, 3 and 4, there were only small discrepancies in the UIV with a maximum of two spinal levels. Small discrepancies of one to two spinal levels were also documented in Lenke 2, 3, and 4 cases. The strongest deviations in the LIV were obtained in Lenke 1 with up to four spinal levels.

**Fig. 4:**
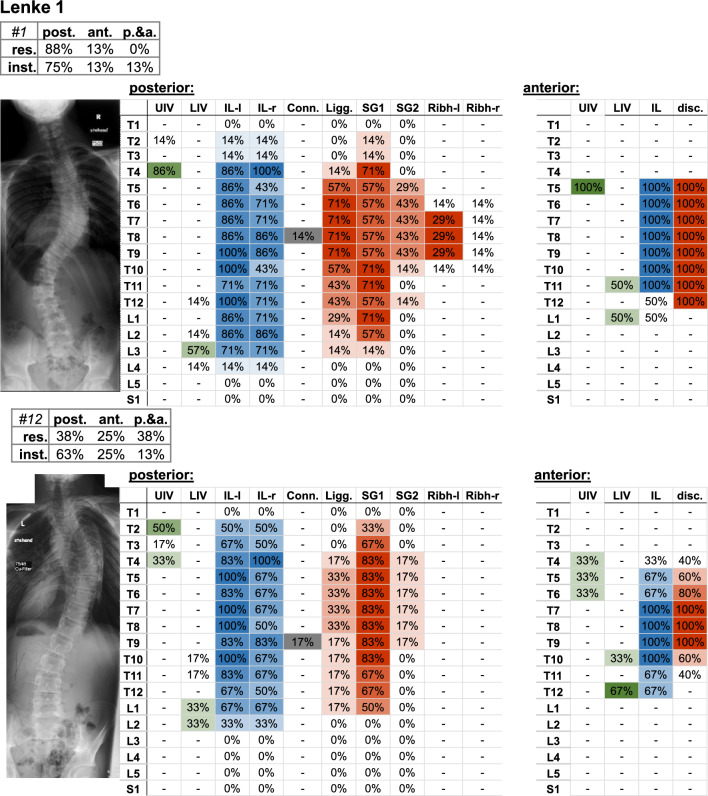
Frontal radiograph and percentage of surgeons who selected the upper (UIV) and lower (LIV) instrumented vertebrae of the Lenke 1 cases. The upper left table indicates the percentage of surgeons (%os) who planned posterior (post.), anterior (ant.), or posterior and anterior (p.&a.) resections (res.) and instrumentation (inst.). Further parameters include the upper (UIV) and lower (LIV) instrumented vertebra, as well as the percentage of instrumented levels on the left (IL-l) and right (IL-r) side, the used connector (Conn.), resected ligaments (Ligg.), Schwab grad 1 and 2 (SG 1/2), resected rib head on the left (ribh-L) and right (ribh-R) side, and discectomy (disc.)

**Fig. 5:**
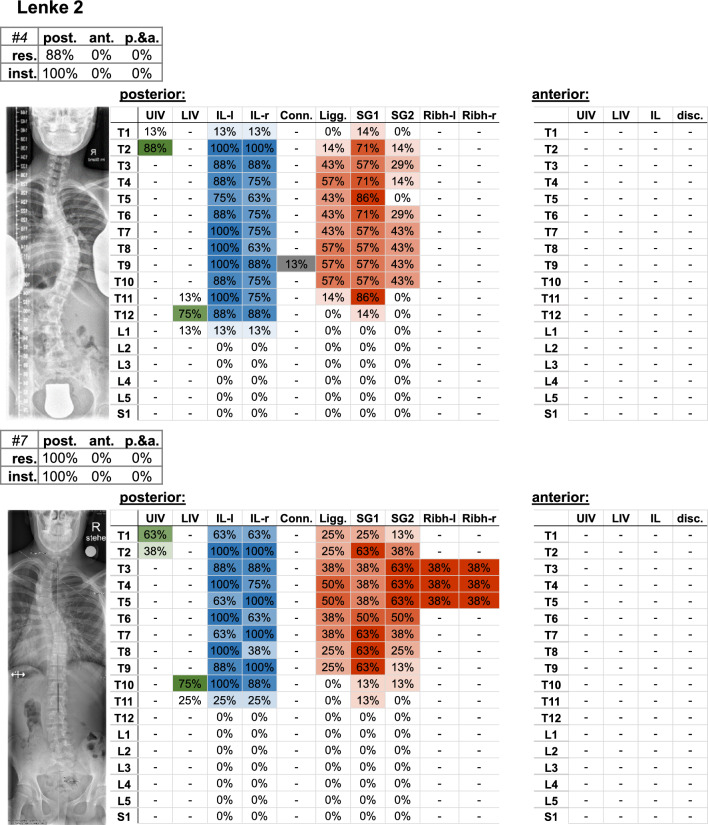
Frontal radiograph and percentage of surgeons who selected the upper (UIV) and lower (LIV) instrumented vertebrae of the Lenke 2 cases. The upper left table indicates the percentage of surgeons (%os) who planned posterior (post.), anterior (ant.), or posterior and anterior (p.&a.) resections (res.), and instrumentation (inst.). Further parameters include the upper (UIV) and lower (LIV) instrumented vertebra, as well as the percentage of instrumented levels on the left (IL-l) and right (IL-r) side, the used connector (Conn.), resected ligaments (Ligg.), Schwab grad 1 and 2 (SG 1/2), resected rib head on the left (ribh-L) and right (ribh-R) side, and discectomy (disc.)

**Fig. 6:**
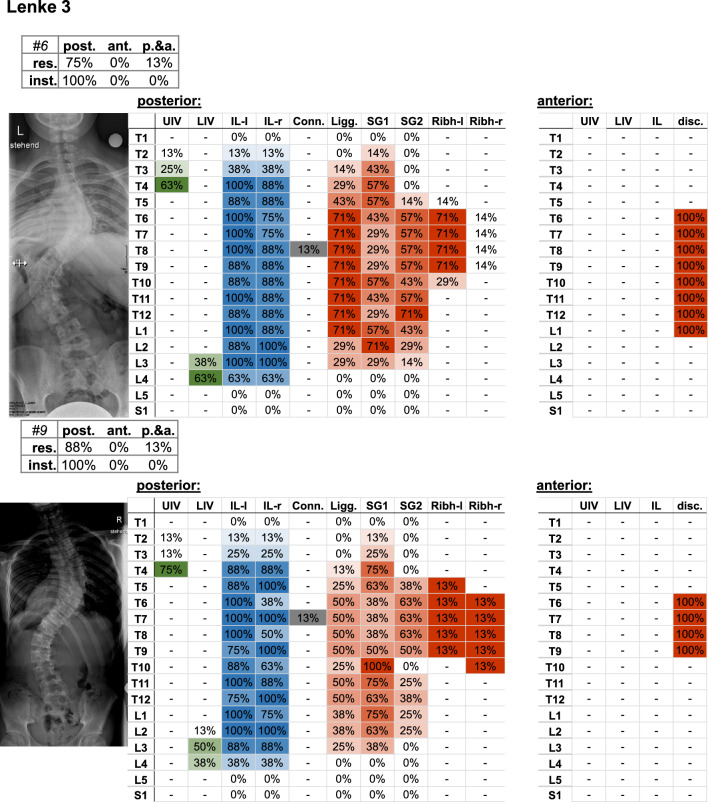
Frontal radiograph and percentage of surgeons who selected the upper (UIV) and lower (LIV) instrumented vertebrae of the Lenke 3 cases. The upper left table indicates the percentage of surgeons (%os) who planned posterior (post.), anterior (ant.), or posterior and anterior (p.&a.) resections (res.) and instrumentation (inst.). Further parameters include the upper (UIV) and lower (LIV) instrumented vertebra, as well as the percentage of instrumented levels on the left (IL-l) and right (IL-r) side, the used connector (Conn.), resected ligaments (Ligg.), Schwab grad 1 and 2 (SG 1/2), resected rib head on the left (ribh-L) and right (ribh-R) side, and discectomy (disc.)

**Fig. 7:**
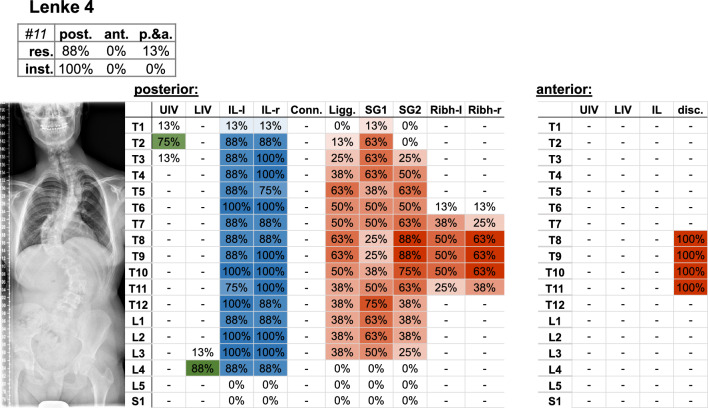
Frontal radiograph and percentage of surgeons who selected the upper (UIV) and lower (LIV) instrumented vertebrae of the Lenke 4 case. The upper left table indicates the percentage of surgeons (%os) who planned posterior (post.), anterior (ant.), or posterior and anterior (p.&a.) resections (res.) and instrumentation (inst.). Further parameters include the upper (UIV) and lower (LIV) instrumented vertebrae, as well as the percentage of instrumented levels on the left (IL-l) and right (IL-r) side, the used connector (Conn.), resected ligaments (Ligg.), Schwab grad 1 and 2 (SG 1/2), resected rib head on the left (ribh-L) and right (ribh-R) side, and discectomy (disc.)

**Fig. 8:**
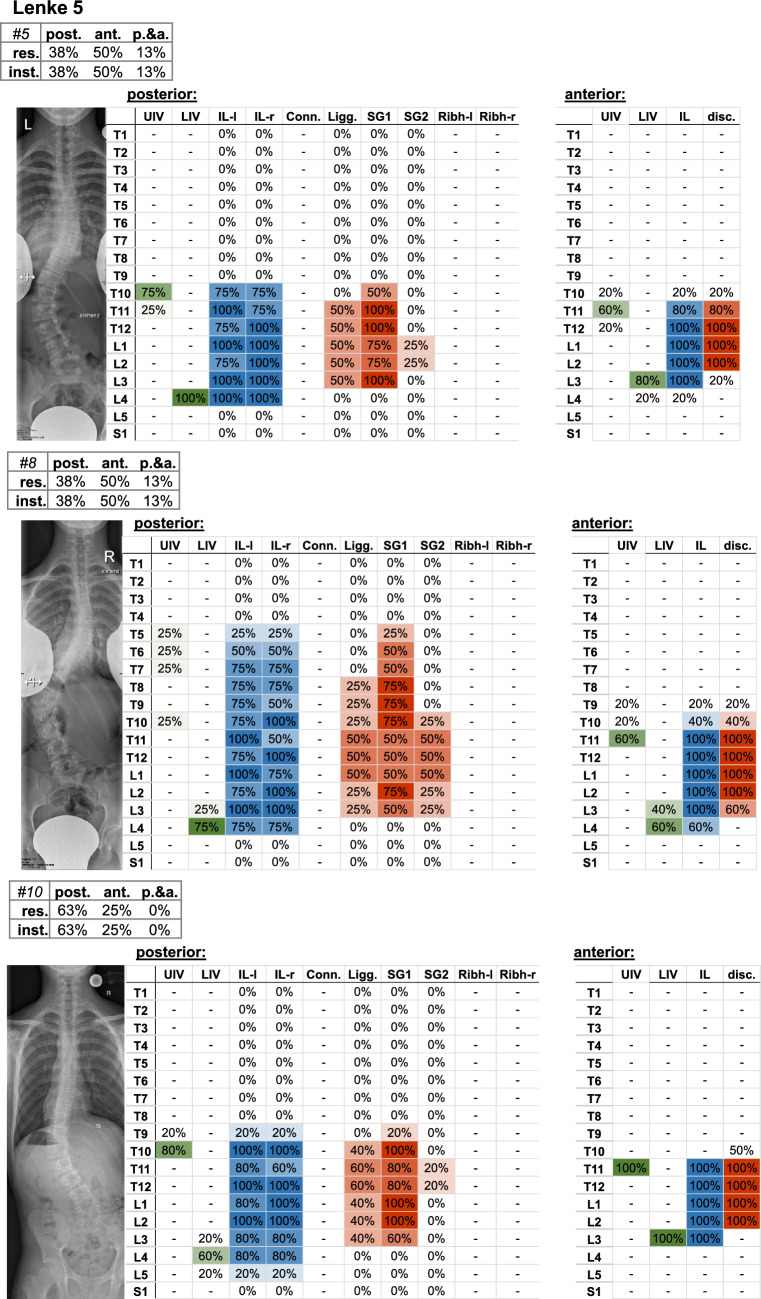
Frontal radiograph and percentage of surgeons who selected the upper (UIV) and lower (LIV) instrumented vertebrae of the Lenke 5 cases. The upper left table indicates the percentage of surgeons (%os) who planned posterior (post.), anterior (ant.), or posterior and anterior (p.&a.) resections (res.) and instrumentation (inst.). Further parameters include the upper (UIV) and lower (LIV) instrumented vertebrae, as well as the percentage of instrumented levels on the left (IL-l) and right (IL-r) side, the used connector (Conn.), resected ligaments (Ligg.), Schwab grad 1 and 2 (SG 1/2), resected rib head on the left (ribh-L) and right (ribh-R) side, and discectomy (disc.)

**Fig. 9:**
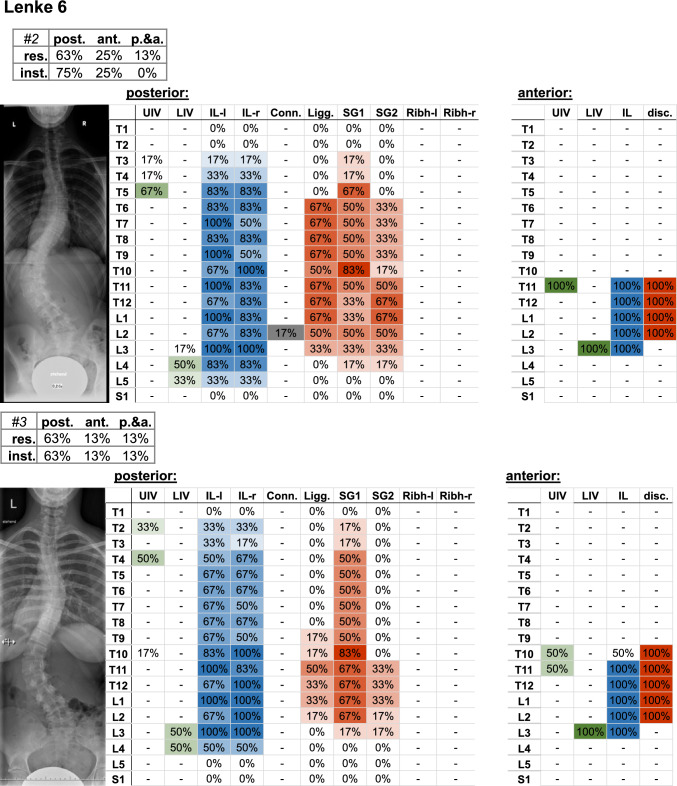
Frontal radiograph and percentage of surgeons who selected the upper (UIV) and lower (LIV) instrumented vertebrae of the Lenke 6 cases. The upper left table indicates the percentage of surgeons (%os) who planned posterior (post.), anterior (ant.), or posterior and anterior (p.&a.) resections (res.) and instrumentation (inst.). Further parameters include the upper (UIV) and lower (LIV) instrumented vertebrae, as well as the percentage of instrumented levels on the left (IL-l) and right (IL-r) side, the used connector (Conn.), resected ligaments (Ligg.), Schwab grad 1 and 2 (SG 1/2), resected rib head on the left (ribh-L) and right (ribh-R) side, and discectomy (disc.)

It should be noted that the amount of deviation within a Lenke curve type may vary between the individual cases (Figs. 4, 5, 6, 7, 8, 9).

### Implant density

The percentage of instrumented levels varied between 67 and 100% of the fused spinal region. The highest average implant density was documented for Lenke 4 and 5 with 94% instrumented levels relative to the overall instrumented length. The lowest implant density was 78% at the right (convex) side of the Lenke 1 main thoracic curvature.

### Used implants

Relative to the overall, the used implants were 70% of polyaxial screws, followed by 19% monoaxial screws. Hooks and cerclage wire were applied only sporadically.

### Further comments of the surgeons

Single comments referred to the shoulder position, which was for some not clearly visible. In one case, the SUK classification system was additionally used. In another case, one surgeon could not perform surgical planning based upon the visible spinal sections within a bending image. Some requested further spinal angles T2–T5 and T10–L2, as well as clinical images.

## Discussion

The surgical strategy of eight experienced scoliosis surgeons on 12 representative AIS cases was documented using a developed questionnaire. The results indicate that commonalities and discrepancies between surgeons depend on the Lenke curve type.

The main discrepancies within the resections steps and fusion length were obtained for Lenke curve types 1, 5, and 6.

### Curve classification

The quantified Fleiss’ Kappa values indicate a good agreement of grouping the AIS cases according to the Lenke classification system [8]. Yet, the presented Fleiss Kappa values were below previously published values [9], indicating a slightly less reliability of the Lenke system. The cause for the reduced inter-rater variability of the Lenke classification system may be implicated by the comments of the surgeons. One surgeon does not use the classification system, as it would be of no help for him. Another would theoretically classify, for instance, a case as a Lenke 5, but would treat it as a Lenke 6. A possible question, therefore, would be whether the specified Lenke classification corresponds to the theoretical or the treated curve type.

### Surgical approach

The posterior approach was planned by all surgeons for Lenke 2, 3, and 4. The overall proportion of surgeons who performed posterior approaches was 76%, which is below the value obtained by De Kleuver et al. (2014), where 96% of the surgeons indicated the posterior approach as optimal [4]. In the same study, 53% of the surgeons indicated the anterior approach as optimal in case of Lenke 5 curve types, which is about the same as that documented here (46%).

### Resection steps/osteotomies

The documented resection steps reveal that osteotomies of Schwab grad 2 are most commonly used. Kleuver et al. also reported that (Ponte-) osteotomies are considered optimal in some cases, particularly at large structural curvatures [4]. Facetectomies in combination with flavectomies were considered optimal at long rigid curves by 73% of the surgeons, which corresponds to the findings within this study.

As presented in Kleuver et al., anterior releases were planned only in some cases, with an overall occurrence of 28%.

### Fusion length

Deviations within the UIV and LIV between surgeons can be referred to the consideration whether to instrument additionally the secondary curvature. This is particularly observed for Lenke 1, 5 and 6. Aubin et al. (2007) already obtained a high variability in the fusion length within a small group of surgeons [2]. Robitaille et al. also observed the lowest consensus between surgeons in the fusion length at Lenke 5 types [12].

Within a single centre study, Erken et al. documented a variability of 31% within four surgeons, who evaluated 100 AIS cases [5]. Most variability was observed for the selection of the UIV.

### Implants

Pedicle screws covered 89% of all used implants, while hooks were only 0.1%. In comparison, Aubin et al. reported the use of hooks in 24% and and Robitaille et al. in 9% of the overall used implants. Robitaille et al. reported all hook constructs in 3% of the reported surgical strategies, while in the present study hooks were only used sporadically, with no all hook constructs. The present study is in line with the findings of the AO survey, which reported pedicle screw constructs as the optimal treatment option [4].

### Limitations of the study

The reported instrumentation strategies were planned by surgeons in a controlled environment with limited patient data, since the surgeons did not have access to the patient in person and perform diagnostics. The results, therefore, only reflect the surgical opinion based on the given radiographic images. This limitation was, yet, necessary to standardize and compare the surgical strategies. Additional factors, which may have an effect on the surgical planning, include the activity of the patient, the patient’s history, and desire. Furthermore, some surgeons mentioned that the surgical strategy may change during surgery, depending on the in situ condition of the patient.

### General remark

In general, the surgical treatment of AIS patients is considered as reliable with low complications [1, 11]. Since surgical treatment is irreversible and complications may occur decades later, it is important to understand the biomechanical influence of the surgery.

Some open questions that can be deduced from the survey: How many resections need to be performed to mobilize the spine efficiently and, at the same time, retain its integrity? What is the optimal spinal fusion length, particularly for Lenke 1, 5 and 6?

## Conclusion

In this survey, the basic parameters for the surgical approach could be documented. Variation in the surgical strategy depended on the Lenke curve type, the direction of the surgical approach, and the surgeon. Main discrepancies included the selection of the UIV of Lenke 5 and 6, as well as the LIV of Lenke 1. Consistencies within the surgeons was documented for the fusion lengths in Lenke 2, 3 and 4 curve types.

The documented discrepancies indicate where open questions in the surgical treatment and the understanding of the biomechanics of scoliosis exist.

## Data Availability

The data that support the findings of this study are not openly available due to reasons of sensitivity and are available from the corresponding author upon reasonable request.
